# Supplementary material for: Modeling glioblastoma heterogeneity as a dynamic network of cell states
Source: Mol Syst Biol. 2021 Sep 16;17(9):e10105. doi: 10.15252/msb.202010105 (PMC8444284; doi:10.15252/msb.202010105)
Supplement: Supplementary file 6 — Source Data for Figure 5 [file MSB-17-e10105-s004.zip › Figure5A_sourcedata/GSEA_3017/nosigdb_stateA.GseaPreranked.1622795026485/gsea_report_for_na_pos_1622795026485.html]

Report for na\_pos 1622795026485 [GSEA]

| GS  follow link to MSigDB | GS DETAILS | SIZE | ES | NES | NOM p-val | FDR q-val | FWER p-val | RANK AT MAX | LEADING EDGE || 1 | GAROFANO\_IAVARONE\_PPR\_SUBTYPE | Details ... | 119 | 0.50 | 3.98 | 0.000 | 0.000 | 0.000 | 549 | tags=99%, list=56%, signal=196% |
| 2 | DOROSZKO\_U3013MG\_CLUSTER\_2 | Details ... | 119 | 0.50 | 3.95 | 0.000 | 0.000 | 0.000 | 386 | tags=76%, list=39%, signal=110% |
| 3 | DOROSZKO\_U3054MG\_CLUSTER\_4 | Details ... | 148 | 0.47 | 3.90 | 0.000 | 0.000 | 0.000 | 562 | tags=97%, list=57%, signal=191% |
| 4 | DOROSZKO\_U3180MG\_CLUSTER\_3 | Details ... | 142 | 0.45 | 3.67 | 0.000 | 0.000 | 0.000 | 482 | tags=86%, list=49%, signal=144% |
| 5 | RICHARDS\_NOWAKOWSKI\_SCIENCE\_2017\_MGE\_DIV\_UPREG | Details ... | 161 | 0.42 | 3.40 | 0.000 | 0.000 | 0.000 | 477 | tags=81%, list=48%, signal=131% |
| 6 | RICHARDS\_NOWAKOWSKI\_SCIENCE\_2017\_MGE\_IPC1\_UPREG | Details ... | 164 | 0.40 | 3.33 | 0.000 | 0.000 | 0.000 | 477 | tags=79%, list=48%, signal=127% |
| 7 | RICHARDS\_NOWAKOWSKI\_SCIENCE\_2017\_IPC\_DIV1\_UPREG | Details ... | 182 | 0.38 | 3.31 | 0.000 | 0.000 | 0.000 | 447 | tags=74%, list=45%, signal=111% |
| 8 | RICHARDS\_ZHONG\_NATURE\_2018\_NPCS\_UPREG | Details ... | 73 | 0.48 | 3.27 | 0.000 | 0.000 | 0.000 | 386 | tags=78%, list=39%, signal=119% |
| 9 | SEURAT\_CELLCYCLE | Details ... | 49 | 0.53 | 3.27 | 0.000 | 0.000 | 0.000 | 453 | tags=94%, list=46%, signal=165% |
| 10 | RICHARDS\_NOWAKOWSKI\_SCIENCE\_2017\_IPC\_DIV2\_UPREG | Details ... | 206 | 0.36 | 3.22 | 0.000 | 0.000 | 0.000 | 477 | tags=74%, list=48%, signal=114% |
| 11 | FELDMAN\_2020\_G1 | Details ... | 85 | 0.42 | 3.02 | 0.000 | 0.000 | 0.000 | 283 | tags=62%, list=29%, signal=80% |
| 12 | LARSSON\_STATE\_4 | Details ... | 57 | 0.47 | 3.01 | 0.000 | 0.000 | 0.000 | 153 | tags=46%, list=15%, signal=51% |
| 13 | RICHARDS\_NOWAKOWSKI\_SCIENCE\_2017\_MGE\_IPC2\_UPREG | Details ... | 157 | 0.34 | 2.89 | 0.000 | 0.000 | 0.000 | 386 | tags=64%, list=39%, signal=88% |
| 14 | SUVA\_G2M\_PMID\_31327527 | Details ... | 30 | 0.53 | 2.79 | 0.000 | 0.000 | 0.000 | 263 | tags=70%, list=27%, signal=92% |
| 15 | RICHARDS\_NOWAKOWSKI\_SCIENCE\_2017\_RG\_EARLY\_UPREG | Details ... | 121 | 0.36 | 2.77 | 0.000 | 0.000 | 0.000 | 165 | tags=38%, list=17%, signal=40% |
| 16 | RICHARDS\_NEFTEL\_CELL\_2019\_G2\_M | Details ... | 30 | 0.53 | 2.76 | 0.000 | 0.000 | 0.000 | 263 | tags=70%, list=27%, signal=92% |
| 17 | DOROSZKO\_U3180MG\_CLUSTER\_4 | Details ... | 46 | 0.45 | 2.74 | 0.000 | 0.000 | 0.000 | 188 | tags=50%, list=19%, signal=59% |
| 18 | NB\_KI\_CLUSTER5 | Details ... | 43 | 0.46 | 2.70 | 0.000 | 0.000 | 0.000 | 477 | tags=88%, list=48%, signal=163% |
| 19 | FELDMAN\_2020\_G2-M | Details ... | 134 | 0.33 | 2.57 | 0.000 | 0.000 | 0.001 | 275 | tags=52%, list=28%, signal=63% |
| 20 | FELDMAN\_2020\_S-G2 | Details ... | 140 | 0.28 | 2.33 | 0.000 | 0.000 | 0.009 | 315 | tags=54%, list=32%, signal=68% |
| 21 | DALMO\_BARCODENUMBER\_UNPUB |  | 102 | 0.29 | 2.22 | 0.000 | 0.001 | 0.019 | 165 | tags=38%, list=17%, signal=41% |
| 22 | GAROFANO\_IAVARONE\_LIST\_OF\_NEURAL\_PROGENITOR\_MARKER\_GENES |  | 36 | 0.40 | 2.18 | 0.003 | 0.001 | 0.024 | 93 | tags=36%, list=9%, signal=38% |
| 23 | DOROSZKO\_U3054MG\_CLUSTER\_6 |  | 33 | 0.37 | 2.08 | 0.003 | 0.002 | 0.059 | 475 | tags=82%, list=48%, signal=152% |
| 24 | RICHARDS\_CAHOY\_JNEUROSCI\_2010\_ASTRO\_YOUNG |  | 173 | 0.22 | 1.93 | 0.000 | 0.007 | 0.178 | 275 | tags=44%, list=28%, signal=50% |
| 25 | RICHARDS\_NOWAKOWSKI\_SCIENCE\_2017\_U2\_UPREG |  | 291 | 0.20 | 1.84 | 0.000 | 0.013 | 0.302 | 310 | tags=44%, list=31%, signal=45% |
| 26 | IVY\_CT |  | 79 | 0.26 | 1.84 | 0.007 | 0.013 | 0.310 | 217 | tags=41%, list=22%, signal=48% |
| 27 | FELDMAN\_2020\_S |  | 62 | 0.28 | 1.81 | 0.009 | 0.014 | 0.344 | 446 | tags=74%, list=45%, signal=127% |
| 28 | RICHARDS\_NOWAKOWSKI\_SCIENCE\_2017\_RG\_DIV1\_UPREG |  | 327 | 0.19 | 1.77 | 0.000 | 0.019 | 0.437 | 323 | tags=43%, list=33%, signal=43% |
| 29 | LARSSON\_STATE\_3 |  | 25 | 0.34 | 1.67 | 0.019 | 0.033 | 0.648 | 101 | tags=36%, list=10%, signal=39% |
| 30 | SUVA\_OPC\_PMID\_31327527 |  | 16 | 0.37 | 1.50 | 0.068 | 0.083 | 0.940 | 276 | tags=56%, list=28%, signal=77% |
| 31 | RICHARDS\_NEFTEL\_CELL\_2019\_OPC |  | 16 | 0.37 | 1.49 | 0.069 | 0.082 | 0.942 | 276 | tags=56%, list=28%, signal=77% |
| 32 | NB\_KI\_CLUSTER3 |  | 15 | 0.37 | 1.48 | 0.074 | 0.086 | 0.954 | 482 | tags=80%, list=49%, signal=154% |
| 33 | RICHARDS\_NOWAKOWSKI\_SCIENCE\_2017\_MGE\_RG2\_UPREG |  | 104 | 0.19 | 1.48 | 0.026 | 0.083 | 0.954 | 472 | tags=65%, list=48%, signal=112% |
| 34 | RICHARDS\_NOWAKOWSKI\_SCIENCE\_2017\_RG\_DIV2\_UPREG |  | 289 | 0.15 | 1.40 | 0.029 | 0.118 | 0.993 | 116 | tags=18%, list=12%, signal=14% |
| 35 | RICHARDS\_ZHONG\_NATURE\_2018\_OPC\_UPREG |  | 31 | 0.25 | 1.30 | 0.173 | 0.189 | 1.000 | 41 | tags=19%, list=4%, signal=20% |
| 36 | RICHARDS\_NOWAKOWSKI\_SCIENCE\_2017\_OPC\_UPREG |  | 90 | 0.17 | 1.25 | 0.167 | 0.233 | 1.000 | 281 | tags=39%, list=28%, signal=49% |
| 37 | DOROSZKO\_U3054MG\_CLUSTER\_0 |  | 20 | 0.27 | 1.22 | 0.233 | 0.254 | 1.000 | 36 | tags=15%, list=4%, signal=15% |
| 38 | PARADA\_T2\_UP\_26461091 |  | 28 | 0.22 | 1.11 | 0.299 | 0.377 | 1.000 | 445 | tags=71%, list=45%, signal=126% |
| 39 | OLSEN\_ADRENERGIC |  | 19 | 0.25 | 1.11 | 0.320 | 0.379 | 1.000 | 264 | tags=42%, list=27%, signal=56% |
| 40 | SINGH\_SOX2\_UPREGULATED\_TRANSCRIPTS\_PMID\_28122245 |  | 77 | 0.15 | 1.04 | 0.386 | 0.461 | 1.000 | 109 | tags=18%, list=11%, signal=19% |
| 41 | RICHARDS\_VERHAAK\_CANCERCELL\_2010\_PRONEURAL |  | 22 | 0.23 | 1.03 | 0.414 | 0.466 | 1.000 | 276 | tags=45%, list=28%, signal=62% |
| 42 | PARADA\_T1\_UP\_26461091 |  | 31 | 0.19 | 0.98 | 0.461 | 0.529 | 1.000 | 243 | tags=42%, list=25%, signal=54% |
| 43 | VANGRONINGEN\_ADRN |  | 59 | 0.14 | 0.89 | 0.629 | 0.671 | 1.000 | 434 | tags=56%, list=44%, signal=94% |
| 44 | NB\_KI\_CLUSTER14 |  | 36 | 0.15 | 0.85 | 0.704 | 0.711 | 1.000 | 151 | tags=22%, list=15%, signal=25% |
| 45 | NB\_KI\_CLUSTER8 |  | 18 | 0.19 | 0.83 | 0.637 | 0.723 | 1.000 | 284 | tags=39%, list=29%, signal=54% |
| 46 | RICHARDS\_NOWAKOWSKI\_SCIENCE\_2017\_NEN\_EARLY1\_UPREG |  | 75 | 0.11 | 0.77 | 0.820 | 0.786 | 1.000 | 364 | tags=45%, list=37%, signal=66% |
Table: Gene sets enriched in phenotype **na **[plain text format]****
